# One-Step Synthesis of a Binder-Free, Stable, and High-Performance Electrode; Cu-O|Cu_3_P Heterostructure for the Electrocatalytic Methanol Oxidation Reaction (MOR)

**DOI:** 10.3390/nano13071234

**Published:** 2023-03-30

**Authors:** Alina Yarmolenko, Bibhudatta Malik, Efrat Shawat Avraham, Gilbert Daniel Nessim

**Affiliations:** The Department of Chemistry and Institute of Nanotechnology, Bar-Ilan University, Ramat Gan 52900, Israel

**Keywords:** heterostructure, synergistic effect, chemical vapor deposition, methanol oxidation reaction, self-standing electrode

## Abstract

Although direct methanol fuel cells (DMFCs) have been spotlighted in the past decade, their commercialization has been hampered by the poor efficiency of the methanol oxidation reaction (MOR) due to the unsatisfactory performance of currently available electrocatalysts. Herein, we developed a binder-free, copper-based, self-supported electrode consisting of a heterostructure of Cu_3_P and mixed copper oxides, i.e., cuprous–cupric oxide (Cu-O), as a high-performance catalyst for the electro-oxidation of methanol. We synthesized a self-supported electrode composed of Cu-O|Cu_3_P using a two-furnace atmospheric pressure–chemical vapor deposition (AP-CVD) process. High-resolution transmission electron microscopy analysis revealed the formation of 3D nanocrystals with defects and pores. Cu-O|Cu_3_P outperformed the MOR activity of individual Cu_3_P and Cu-O owing to the synergistic interaction between them. Cu_3_P|Cu-O exhibited a highest anodic current density of 232.5 mAcm^−2^ at the low potential of 0.65 V vs. Hg/HgO, which is impressive and superior to the electrocatalytic activity of its individual counterparts. The formation of defects, 3D morphology, and the synergistic effect between Cu_3_P and Cu-O play a crucial role in facilitating the electron transport between electrode and electrolyte to obtain the optimal MOR activity. Cu-O|Cu_3_P shows outstanding MOR stability for about 3600 s with 100% retention of the current density, which proves its robustness alongside CO intermediate.

## 1. Introduction

The constant rise in global energy demand with the simultaneous depletion of the existing fossil fuels of the earth crust are driving the research of new energy sources [1,2,3,4]. To resolve such issues of climate change and the energy crisis, direct methanol fuel cells (DMFCs) have met tremendous interest owing to their potential application in both mobile and stationary devices [5,6,7]. DMFCs have several advantages, including being simple to operate and the fact that methanol is nontoxic and can be stored and transported very easily. More importantly, they exhibit a capacity than can deliver high power density [8,9]. Usually, the electro-oxidation steps of methanol follow several pathways that are coupled with protons and electrons. The methanol oxidation reaction (MOR) that occurs at the anode converts the chemical energy present within the methanol to electricity: CH_3_OH + 6OH^−^ → CO_2_ + 5H_2_O + 6e^−^ (At pH = 14, E^0^ = −0.81 V NHE) [10]. The major problems of this reaction are the slow kinetics of MOR (multielectron transfer process) and the poisoning of the catalyst due to the formation of C-O at the catalytic active sites, thus hampering the practical use of DMFCs [11,12,13,14]. Operating DMFCs in alkaline media can be preferred over acidic media owing to the beneficial effects of obtaining higher efficiency, the utilization of non-noble and cheaper metals, and a lower rate of harmful effects exhibited by intermediates on the catalytic active sites of catalysts [15,16]. It is found that the successful accomplishment of the direct methanol-based fuel cell technology mainly relies on two key parts, such as the membrane and the electrocatalysts (anodes). DMFCs face two major challenges, namely (a) the methanol crossover effect, which can be sorted out by developing efficient membranes; and (b) slow MOR kinetics occurring at the anode. Thus in this scenario, efficient and high-performance anode catalysts could facilitate the reaction kinetics and improve MOR activity [17]. These two major issues not only frustrate to the cathode output, but also reduce the fuel efficiency. Notably, the anodic MOR occurs in alkaline media comparatively faster than it does in acidic media. Moreover, the kinetics of cathodic ORR is known to be more appealing in alkaline media than in acidic media [18].

Noble metals such as Pt, Pd, and their alloys provide outstanding MOR performance in both acidic and basic electrolytic conditions [19,20,21,22,23,24,25,26]. Platinum is a traditional metal that is used for DMFCs as an anode catalyst, and outperforms other studied metals in terms of activity and stability. However, the formation of intermediates such as carbon monoxide (CO) while oxidizing the methanol blocks the active site of catalysts, and hence lowers the methanol electro-oxidation reaction kinetics through the catalyst poisoning, which is a roadblock of the DMFC industrialization [27]. Among the Pt-based binary alloys, Pt–Ru alloy shows promising behavior and is considered the state-of-the-art anode catalyst for DMFCs [28,29]. Nevertheless, Pt and their alloy catalysts are highly expensive, which thus restricts their industrial feasibility in DMFCs [30,31]. Compared to Pt, the Pd-based catalysts have certain advantages, such as the abundance of Pd being much higher (0.015 ppm) than Pt (0.005 ppm) in the Earth’s crust, which can reduce the catalyst price. Moreover, Pd-based catalysts exhibit good corrosion stability, and operate well even in alkaline conditions, which makes the commercialization of alkaline DMFCs possible [32].

In contrast to noble metal catalysts, non-noble metals are abundant, inexpensive substitutes to their platinum group metal counterparts for the MOR applications. Furthermore, nonprecious-metal-based catalysts are the best choices to operate in alkaline DMFCs. In this regard, first-row transition metal-based oxides and hydroxides are possible substitutes owing to their exciting properties and electrocatalytic activity [33,34,35,36]. In recent decades, transition metals, comprising of Ni, Co, and Cu, as well as their compounds, have gained tremendous research attention [37]. Among the other first-row *d*-block metals, copper-based systems are promising owing to their high catalytic performance, low price, and nontoxicity [11,38,39,40,41].

Moreover, heterostructures can exhibit much higher electrocatalytic activity compared to their single-component counterparts [42]. In recent years, studies have found that multicomponent systems are capable of lowering the binding strength between metal and intermediates, thus delivering a surplus of hydroxyl ions, which is useful for the oxidation of intermediates [43]. The multicomponent heterostructures usually act in balancing the adsorption and desorption of formed chemical intermediates. Mostly, electrocatalytic reactions occur at the interfaces of the electrode and electrolyte, hence the electronic environment and the surface properties of a catalyst decide their electrocatalytic performance. In this regard, integrating two different components by means of forming heterointerfaces could boost electrocatalytic activity [44,45]. The catalysts are usually immobilized on current collectors with the help of polymeric binders prior to the testing. However, the polymer binders enhance the solution resistance, sometimes causing the blockage of the catalytic active sites, leading to reduced catalytic performance [46]. In this aspect, the direct growth of materials over conductive support has gained support in recent years, as self-supported electrodes do not require binders (which increase the electrical resistance and affect long-term stability) and deliver a very good output with low loading of materials [32,47]. Next-generation DMFC anode catalysts must exhibit high performance, good durability character, and cost effectiveness. 

Inspired by the concept of interface engineering, we successfully engineered Cu-O|Cu_3_P heterostructures as “self-supported” electrodes using a two-zone atmospheric pressure chemical vapor deposition (AP-CVD) furnace. The binder-free Cu-O|Cu_3_P anode unveiled promising MOR activity and stability in 1 M CH_3_OH containing 1 M KOH. It also showed good conductivity and high tolerance against CO, the intermediate forms during MOR. 

## 2. Experimental Section

**Scheme 1:**
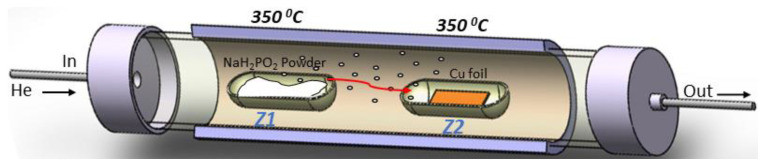
The drawing demonstrates the synthesis method of Cu-O|Cu_3_P.

Commercial copper foils (99.9% purity, 0.254 mm thickness) of about 1 × 3 cm^2^ were used as the starting material. The copper pieces were cleaned with 1 M HCl for 20 min, followed by rinsing with deionized water and drying with N_2_-gas flow. We synthesized Cu-O|Cu_3_P using a two-furnace atmospheric pressure chemical vapor deposition (AP-CVD) furnace. A fused silica (quartz) tube with an internal diameter of 22 mm was inserted in the two furnaces in series. The temperatures of the furnace were monitored using built-in thermocouples. The temperatures were set at 350 °C for both the upstream (Z_1_) and downstream furnaces (Z_2_). In the first step, we oxidized the Cu under atmospheric pressure (kept at Z_2_) with a temperature ramp of 5 °C min^−1^ at 350 °C for an hour to obtain Cu-O. In the second step, after oxidation, we purged the system with He gas 100 [sccm] (99.9999% gas technologies) for 30 min. Finally, we pushed the alumina boat containing NaH_2_PO_2_·H_2_O powder (100 mg, Glentham) into the hot upstream furnace (Z_1_) under He flows (for 60 min., 100 sccm) followed by a slow natural cooling of the furnace (depicted in Scheme 1). Similarly, we carried out the oxidation process of Cu foil at 500 °C with the increase in temperature at 5 °C min^−1^ and held for 1 h, and in the next step, we placed a boat containing 100 mg of NaH_2_PO_2_·H_2_O powder in the in the upstream zone (Z_1_) of the preheated furnace (at 350 °C) under He flows. Then, the furnace was cooled naturally to room temperature.

## 3. Materials Characterization

The synthesized materials on Cu foil were characterized using X-ray diffraction (XRD) (Bruker D8 (Billerica, MA, USA) advance with Cu Kα radiation (λ = 1.5418 Å) with an operating voltage of 40 kV). The morphological and microstructural analyses were performed using a high-resolution scanning electron microscope (HR-SEM) and a high-resolution transmission electron microscope (HR-TEM). For the HR-SEM analysis, FEI, Magellan 400L (Hillsboro, OR, USA) was utilized to obtain the morphology and topography. HR-TEM analysis was carried in (JEOL JEM-2100, Tokyo, Japan). Before the TEM analysis, we dispersed 0.5 mg of the sample (scratched from Cu foil) in 15 mL absolute ethanol, and then we drop-casted a drop of solution onto Ni grid and kept it for drying at ambient conditions. The Kratos axis HS spectrometer (Kratos, San Diego, CA, USA), combined with monochromatic (Al) and dual (Mg/Al) source, was utilized for the XPS analysis. We attached a pinch of sample to the sticky carbon tape for the measurement.

## 4. Electrochemical Characterization

Electrochemical experiments were performed in a standard three-electrode system using a Biologic VSP potentiostat. Pt and Hg/HgO (filled with 1 M NaOH) served as counter and reference electrodes, respectively. The nanostructured catalysts grown over Cu foil were directly used as the working electrodes without further treatment. The measured data that were used for the calculation were *iR* drop-corrected (*iR* drop was compensated concerning open-circuit potential). The whole study was conducted in an alkaline medium (1 M KOH as the electrolyte). The electrolytic solution was prepared using millipore water (resistance of 18 MΩ). The data were measured after conducting *I*–*V* polarization of the electrodes for about 100 CV cycles. The electrocatalytic activities were assessed using cyclic voltammetry (CV), chronoamperometry (CA), and electrochemical impedance spectroscopy (EIS) for the prepared catalysts. 

## 5. Results and Discussion

**Figure 1:**
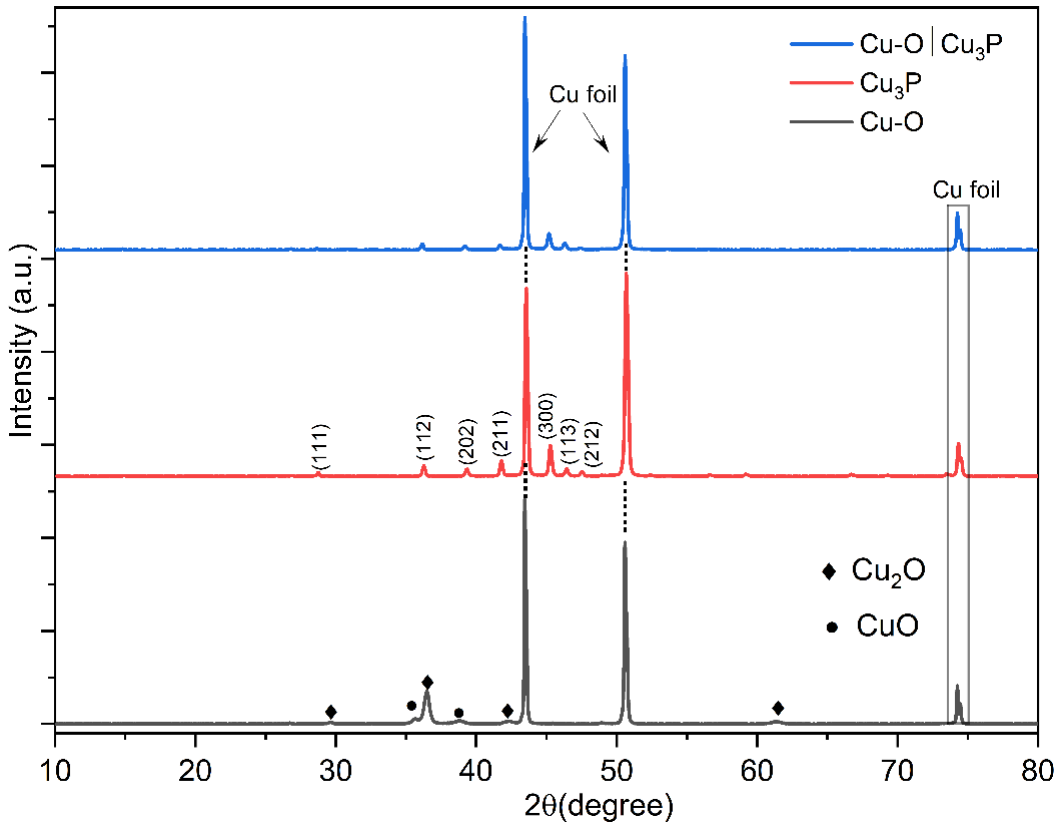
The powder XRD of Cu-O, Cu_3_P, and Cu_3_P|Cu-O.

We obtained the Cu-O, Cu_3_P, and Cu-O|Cu_3_P grown over Cu foil using an AP-CVD system at an optimized temperature of 350 °C (optical images are provided in Figure S2) and characterized them using various physical characterization techniques. Evidently, when the reaction temperature at Z2 exceeded 350 °C, the formed products started cracking from the Cu foils (as given in Figure S1), leading to the nonuniform distribution of active catalysts. The product ended up with substantial fractures and gaps when the synthesis was carried out at 500 °C, which prevented us from using these as free-standing electrodes; however, we could use them in the form of powder catalysts. The powder pattern XRD was used to identify the phase purity and crystallinity of the synthesized materials. Figure 1 confirms that the materials that were grown over Cu foil are highly crystalline in nature. After oxidation of the Cu foil, we observed that the diffraction peaks present at 2 θ of 29.6°, 36.5°, 42.2°, and 61.3°, belong to the (110), (111), (200), and (220) planes of Cu_2_O (reference code; 05-0667) respectively [48]. Further, the existence of CuO was confirmed, as the peaks located at 2 θ of 35.5° and 38.7° fit to the (002) and (111) planes of CuO (reference code; 45-0937). This indicates that mixed oxides such as Cu_2_O and CuO were grown over Cu foil, which we termed Cu-O. The crystal planes of the as-formed Cu_3_P were confirmed by indexing the existing peaks of Cu_3_P to their corresponding (*hkl*) planes (JCPDS No. 02-1263) [49]. In all cases, the peaks present at 43.45, 50.5 and 74.2° belong to metallic Cu (due to Cu foil). The diffraction pattern (in blue color) contained peaks of Cu-O and Cu_3_P with slight peak shifting towards lower/higher 2 θ values, as shown in Figure 1.

**Figure 2:**
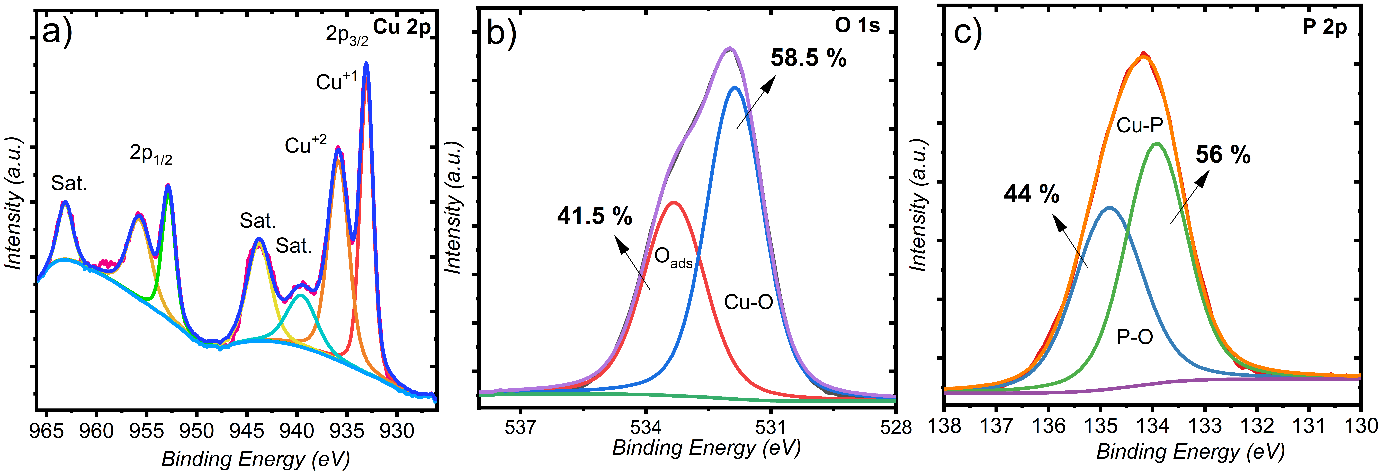
Core-level high-resolution XPS spectra of (**a**) Cu 2p; (**b**) O 1s due to the existence of CuO; and (**c**) P 2p due to the presence of Cu_3_P.

X-ray photoelectron spectroscopy (XPS) analysis was used to examine the formed hybrid nanostructure’s chemical states and surface nature (Table S1). Figure 2a stands for the 2p high-resolution XP spectrum of Cu 2p. The peaks located at the binding energy positions of 933.1 eV belong to Cu 2p_3/2_ of Cu(I), which could be due to the formation of Cu_3_P and Cu_2_O. The peaks present at 935.5 eV under Cu 2p_3/2_ can be assigned to the presence of Cu (II). Two satellite peaks at 939.8 eV and 943.7 eV can be attributed as CuO and Cu_3_P, respectively. The O 1s spectrum in Figure 2b indicates that the peaks that arise at 531.8 eV and 533.2 eV can be ascribed to Cu-O bonding and adsorption of oxygen on the Cu_3_P surface, respectively. As shown in Figure 2c (P 2p spectrum), the peaks located at the binding energies of 133.8 eV and 134.6 eV belong to Cu-P and P-O bonding correspondingly. Notably, the deconvolution percentage Cu-O is 58.5% while the surface adsorbed oxygen percentage is found to be 41.5%. The evaluated deconvolution percentage of Cu-P bonding is fixed at 56% while 44% of PO_X_ species exists. 

**Figure 3:**
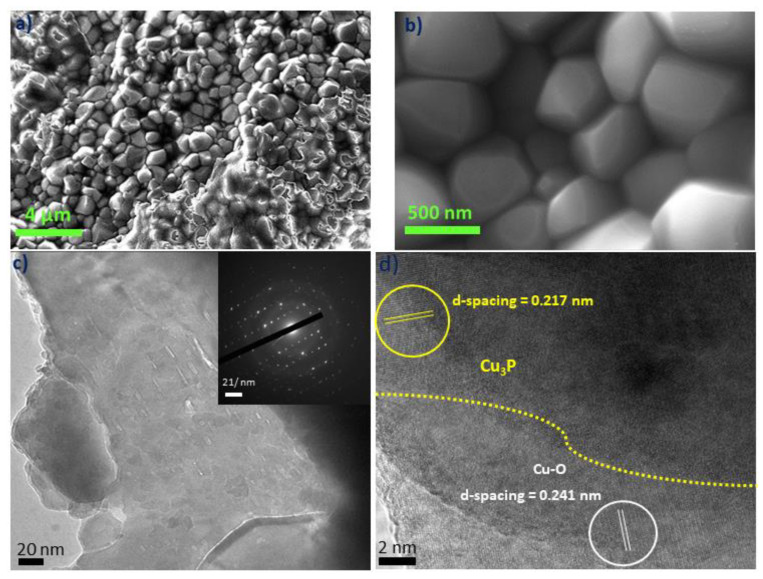
HR-SEM of the formed heterostructure: (**a**) low-resolution image, (**b**) high-resolution image; (**c**) HR-TEM image of Cu-O|Cu_3_P (inset; SAED pattern); and (**d**) lattice distance measurement from high-resolution image.

The chemical composition and morphology of the formed Cu-O|Cu_3_P hetero-structure was examined by high-resolution scanning electron microscopy (HR-SEM) and high-resolution transmission electron microscopy (HR-TEM) (Figure S5). The HR-SEM of both low- and high-resolution images (Figure 3a,b) show the formation of distinct nanocrystals with flat faces. Mostly, the nanocrystals are irregular polygon types, where the edges and corners are a little truncated. At higher resolution, the significant number of pores and voids on the structure can be seen (represented in Figure 3c), confirming the defectiveness of the structure. The selected area diffraction (SAED pattern) reveals that the material is polycrystalline in nature, as provided in the inset of Figure 3c. Figure 3d denotes the lattice fringes of the formed material; the higher *d*-spacing could be indexed to Cu-O and the lower d-spacing value could be due to Cu_3_P. Moreover, the heterointerface was observed in the structure, due to the presence of two different orientations of lattice fringes separated by a border line (which we marked with a yellow dotted line). 

**Figure 4:**
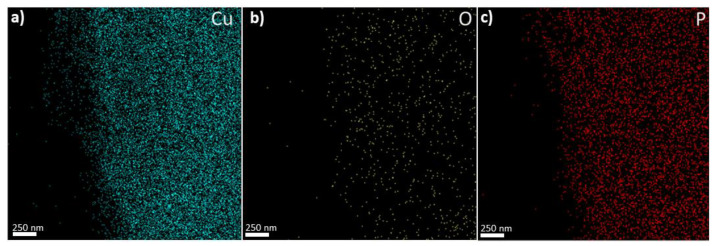
STEM color mapping image of the heterostructure to differentiate the elements (**a**) copper, (**b**) oxygen, and (**c**) phosphorous.

The STEM mapping image shows the distribution of elements (Figure 4), and the individual mapping images show that Cu occupies the whole part of the structure (Figure 4a). O and P are also confined everywhere in the structure, as is Cu (Figure 4b,c). However, the density of phosphorous seems to be greater than oxygen, which indicates that the formation of Cu_3_P is dominated by Cu-O. The HR-TEM of the post-MOR sample is provided in Figure S6 in order to observe the changes. The post-MOR of Cu-O|Cu_3_P was analyzed using HR-TEM. Figure S6a,b shows the low- and high-resolution TEM images, respectively. The morphology resembles a sheet-type structure, as shown in Figure S6a. Significant cracks and wrecks were observed for Cu-O|Cu_3_P (high-resolution image) owing to the strong oxidation environment in the alkaline electrolyte-containing methanol fuel. The EDS in Figure S6c demonstrates the presence of Cu, O, and P (K stems from the electrolyte that we used in the study; KOH). Figure S6d shows that after MOR in alkaline solution, the planes of Cu-O|Cu_3_P are significantly disordered and damaged. 

## 6. Electrocatalytic MOR Study

**Figure 5:**
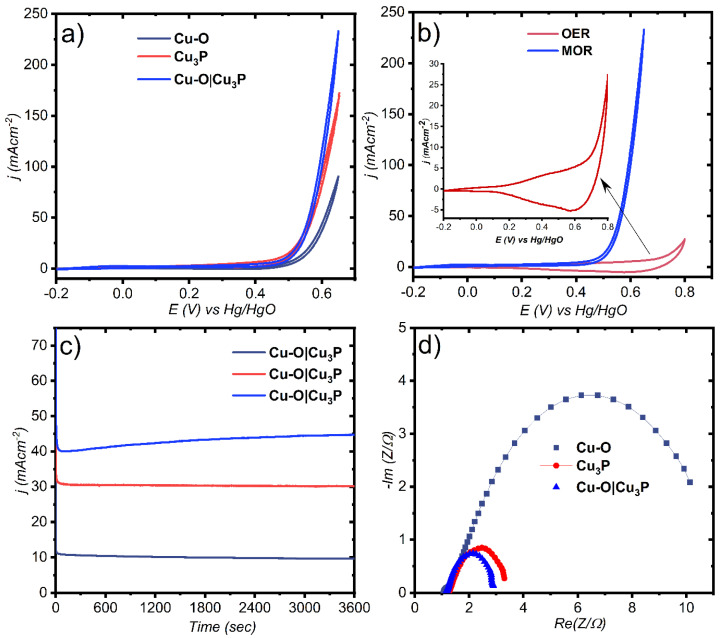
(**a**) Cyclic voltammetry showing MOR of three catalysts carried out in 1 M methanol and 1 M KOH; (**b**) comparative OER and MOR results of Cu-O|Cu_3_P (the inset presents OER result); (**c**) comparison of CA results carried out at fixed faradic potentials; and (**d**) Nyquist impedance plots of Cu-O, Cu_3_P, and Cu-O|Cu_3_P.

The electrocatalytic activity of the synthesized electrodes towards anodic OER and MOR have been studied in both CH_3_OH-free and 1 M CH_3_OH-containing 1 M KOH (pH = 14) electrolytes. Here, we used 1 M CH_3_OH as the fuel, as it is reported that concentrated methanol solution improves the power density of DMFCs and reduces the cell size [50]. We carried out cyclic voltammetry (CV) to probe the catalytic activity at a sweep rate of 20 mVs^−1^. The MOR activities of the three studied catalysts are shown in Figure 5a. Cu-O exhibited a MOR onset potential of 0.51 V vs. Hg/HgO and achieved a current density (*j*) of 10 and 50 mAcm^−2^ at 0.52 V and 0.61 V vs. Hg/HgO, respectively. The Cu_3_P showed a MOR onset potential of 0.47 V vs. Hg/HgO, which is just 10 mV lower than the onset potential observed in the case of Cu-O|Cu_3_P. Cu_3_P showed 50 and 100 mAcm^−2^ at the potential of 0.56 and 0.60 V vs. Hg/HgO. Cu_3_P|Cu-O delivered the *j* of 50 and 100 mAcm^−2^ at 0.55 and 0.58 V vs. Hg/HgO. Further, Cu_3_P|Cu-O shows the maximum anodic current density (*j*) of 232.5 mAcm^−2^ at the vertex potential 0.65 V vs. Hg/HgO, while the *j_max_* for Cu-O and Cu_3_P were observed as 170 and 91 mAcm^−2^, respectively, at the same potential. This confirms that the heterostructure exhibits a tremendous impact on MOR in an alkaline medium. In Figure 5b, we observe that without methanol, Cu-O|Cu_3_P exhibits a high OER onset potential of 0.68 V vs. Hg/HgO with a low current density, which differentiates the performance of OER from MOR. 

We examined the electrocatalytic stability and catalyst poisoning by CO for the electrodes during MOR using chronoamperometry (CA) tests at constant applied potentials. Cu-O showed a significant drop in current density over a period of 3600 s, as shown in Figure 5c; this behavior could be attributed to catalyst poisoning, which indicates poor MOR activity. Similarly, in the case of Cu_3_P, the *j* over time decreased from 32 mAcm^−2^ (at 10 s) to 30.5 mAcm^−2^ (at 3600 s), also indicating low stability against the hazardous intermediates of methanol oxidation. However, the stability behavior exhibited by Cu-O|Cu_3_P showed a quite different phenomenon. The current started increasing up to 2500 s, and then the current density reached a steady value up to 3600 s, without any loss (*j* = 44.5 mAcm^−2^ from 2500–3600 s). The improved stability of Cu_3_P|Cu-O indicates that the combination of Cu_3_P and Cu-O substantially improves the catalyst poisoning tolerance activity. Usually, at the starting of the reaction, each catalyst shows fast kinetics, as the active sites are free from the adsorbed methanol molecules at the surface. Then, methanol molecules adsorb on the electrocatalytic sites via methanol oxidation, or form intermediate species such as CO, CH*_x_*, and CH_2_O after several minutes (assumed as the rate-determining step), which contribute to the catalyst poisoning. The noticeable decrease in the current density could be attributed to the poisoning of the catalysts by hazardous chemical species [50].

Electrochemical impedance spectroscopy (EIS) is a crucial electrochemical measurement technique that helps to identify the kinetic and mass transport phenomena. We carried out EIS measurements in the frequency range of 100 kHz to 100 mHz with an applied amplitude of 10 mV at the faradic potentials (corresponding MOR potentials of 0.57 V vs. RHE). Cu-O Cu_3_P|, Cu_3_P, and Cu-O showed two depressed semicircles without any indication of diffusion phenomena, as shown in the Nyquist plot (Figure 5d). The arc of the semicircle formed by Cu-O Cu_3_P| is quite smaller compared to the semicircles of Cu_3_P and Cu-O, which points to the improved electronic conductivity of Cu-O|Cu_3_P. Semicircles such as the one at higher frequency and another at low frequency are attributed to the presence of two types of resistance and two-time constants (Figure 5d). The equivalent circuit diagram obtained from the fitted Nyquist plot is depicted in Figure S3. The correct way to carry out the impedance simulation is pivotal to obtain accurate data. Necessarily, we replaced the capacitor (C) with the constant phase element (Q) while fitting the data in the equivalent circuit diagram. It is mostly accepted that the presence of Q and the depressed semicircles was observed in cases of solid electrodes due to the surface roughness, and thus C can be only used in ideal cases [51]. In this case, R1, R2, and R3 denote solution resistance, resistance due to the metal oxidation/adsorbed species on the metal surface, and the charge transfer resistance, respectively. The charge transfer resistance in this case signifies the rate at which the charge transfer occurs during MOR (how fast) with changing the electrode potential by the time the surface coverage by the intermediate remains constant. Cu-O|Cu_3_P exhibits a smaller charge transfer resistance of 1.5 ohm, indicating a faster MOR kinetics (details of the fitting parameters are given in Figure S3). 

The electrochemical active surface area (ECSA) of the three materials were evaluated using double-layer capacitance (C_dl_), which we obtained from the cyclic voltammetry (CV) plots in the nonfaradic regions (Figure S4). We measured the CVs for these catalysts in a potential range of −0.2–0 V at scan rates ranging from 20 to 160 mVs^−1^. The calculated C_dl_ for Cu-O, Cu_3_P, and Cu-O|Cu_3_P are 0.4, 1.3, and 5 mFcm^−2^, respectively. ECSA can be obtained from C_dl_/C_s_, where the specific capacitance (Cs) for the flat electrodes is 40 μF cm^−2^. Thus, the ECSAs for Cu-O, Cu_3_P, and Cu-O|Cu_3_P are given as 10, 32.5, and 125 cm^2^, respectively. The roughness factor (RF) can be calculated from ECSA as RF = ECSA/A_geo_ (geometrical area of the substrate which is 1 cm^2^ for all electrodes in this study). Cu-O, Cu_3_P, and Cu-O|Cu_3_P exhibit RFs of 10, 32.5, and 125, respectively. The heterostructure displays the highest double-layer capacitance, ECSA, and RF values, which has good agreement with the MOR catalytic activity. 

The electrocatalytic MOR performance of the Cu-O|Cu_3_P heterostructure that is grown over Cu foil is much better than some earlier reports, such as Cu(OH)_2_−CuO nanoneedle array on the Cu foil [52]; NPC dealloyed by Cu_66_Ti_30_Ni_4_ amorphous ribbon [40], Ni-MgO/C [53], NF-converted NiO/NF [54] hierarchical porous Co_3_O_4_/NiCo_2_O_4_ [55], and ZnCo_2_O_4_ NPs on Ni foam [56], as given in Table 1. Cu_3_P|Cu-O achieves the high current density of 232.5 V at the low potential of 0.65 V vs. Hg/HgO, while other reported self-standing electrodes achieve high current densities at higher potential. 

The high catalytic reactivity of Cu-O|Cu_3_P towards MOR could be attributed to (1) the direct growth of Cu-O|Cu_3_P on Cu foil providing close bonding between substrate and catalyst, excellent adhesion, and effective electrical contact; (2) the defective, 3D pattern of the nanocrystals and synergistic interaction between Cu-O and Cu_3_P facilitating adequate passage of reactants and products; and (3) binder-free strategy further avoiding an unnecessary increase in *i*R drop and catalyst loss during reaction, and thus improving the catalytic activity. Thus, the practical application of DMFCs depends on the development of efficient anode catalysts that efficiently catalyze methanol oxidation reaction. 

## 7. Conclusions

Using a facile synthesis, we fabricated a self-supported electrode comprising a Cu-O| Cu_3_P heterostructure on Cu foil. The integrated architecture demonstrates enhanced electronic conductivity, remarkable electrocatalytic performance towards the electro-oxidation of methanol, and good reaction stability. Specifically, it exhibits a small charge transfer resistance, low MOR onset potential, higher current density, and good MOR diffusion kinetics compared to the individual Cu-based synthesized catalysts. This electrocatalytic performance improvement for Cu-O|Cu_3_P can be credited to the synergistic or interfacial effect exhibited by the heterointerfaces. The abundance of copper and the scalability of the synthesis process, along with the high anodic output, promote its implementation in DMFCs. This study might be useful for developing Cu-based heterostructures or core–shell structures to obtain optimal activity for energy conversion applications.

## Figures and Tables

**Table 1:** MOR performance comparison of the Cu_3_P|Cu-O grown over Cu Foil with other reported free-standing electrodes.

Electrode	Reference Electrode	CH_3_OH Conc. in 1 M KOH	Maximum Anodic Current Density (mAcm^−2^)	Potential (V)
Cu(OH)_2_−CuO/Cu [52]	Hg/HgO	0.5 M	60.2	0.65
NPC-66 [40]	Ag/AgCl	1 M	298	1.2
Ni-MgO/C [53]	Hg/HgO	1 M	192	0.94
NiO/NF [54]	Ag/AgCl	1 M	300	0.82
Co_3_O_4_/NiCo_2_O_4_ [55]	Hg/HgO	0.5 M	140	0.6
ZnCo_2_O_4_ NPs [56]	SCE	1 M	270	0.7
**Cu-O|Cu_3_P (this work)**	**Hg/HgO**	**1 M**	**232.5**	**0.65**

## Data Availability

Data are unavailable due to privacy or ethical reasons.

## Supplementary Materials

The following supporting information can be downloaded at: https://www.mdpi.com/article/10.3390/nano13071234/s1, Figure S1: Optical images of Cu foil heated at 500 °C in air title; Figure S2: Optical images of Cu-O and Cu-O|Cu_3_P grown over Cu foil; Figure S3: The equivalent circuit diagram fitted from the Nyquist plot of Cu-O|Cu_3_P; Figure S4: CVs and the double-layer capacitance plots of three different catalysts; Figure S5: The HR-TEM image of Cu-O|Cu_3_P; Figure S6: Post-MOR HR-TEM of Cu-O|Cu_3_P; Table S1: Representation of the deconvoluted compositions of elements.
