# Supplementary material for: One-Step Synthesis of a Binder-Free, Stable, and High-Performance Electrode; Cu-O|Cu3P Heterostructure for the Electrocatalytic Methanol Oxidation Reaction (MOR)
Source: Nanomaterials (Basel). 2023 Mar 30;13(7):1234. doi: 10.3390/nano13071234 (PMC10096724; doi:10.3390/nano13071234)
Supplement: Supplementary file 1 [file nanomaterials-13-01234-s001.zip › nanomaterials-2293967-supplementary.pdf]

nanomaterials

MDPI

Supplementary Materials

One-Step Synthesis of a Binder-Free, Stable, and High Performance Electrode; Cu-O|Cu_3P Heterostructure for the Electrocatalytic Methanol Oxidation Reaction (MOR)

Alina Yarmolenko †, Bibhudatta Malik †, Efrat Shawat Avraham † and Gilbert Daniel Nessim *

The Department of Chemistry and Institute of Nanotechnology, Bar-Ilan University, Ramat Gan 52900, Israel
* Correspondence: gdnessim@biu.ac.il
† These authors contributed equally to this work.

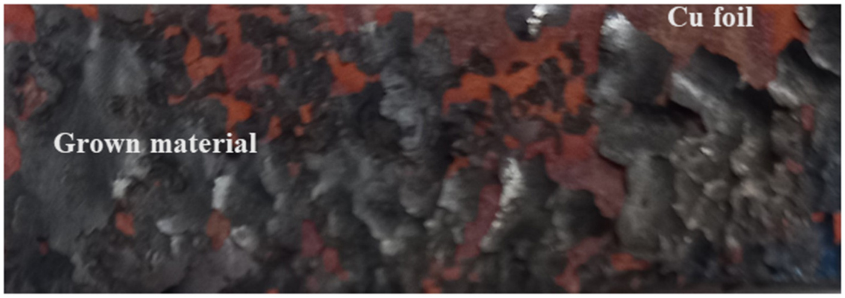

Figure S1. The optical photograph represents the Cu foil heated at 500 °C in air. The material formed with it are found to be ruptured and thus it is not suitable to act as a binder free electrode.

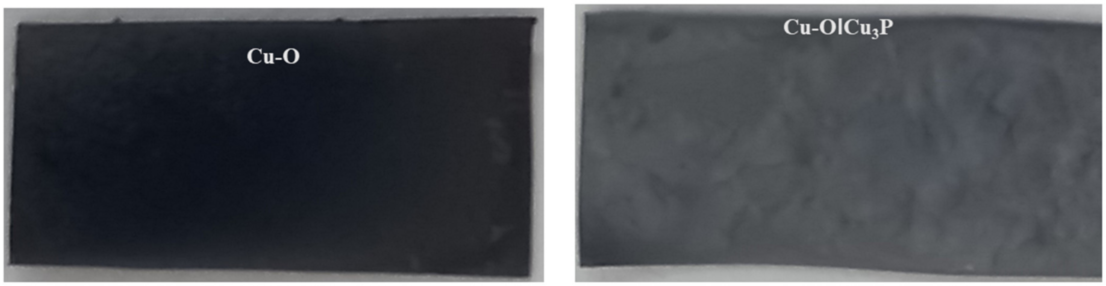

Figure S2. Left image stands for mixed Cu oxides (Cu-O) grown over Cu foil (heated in air at 350 °C) and the right one denotes the optical image of Cu-O|Cu_3P that grown over Cu foil. In both cases the front and rear sides are fully converted to Cu based oxides and phosphide.

Table S1. represent the deconvoluted compositions of elements and C 1s arises from the substrate (carbon tape) while measuring the XPS.

Elements	Atomic percentage
Cu 2p	12.66
P 2p	15.56
O 1s	63.48
C 1s	8.3

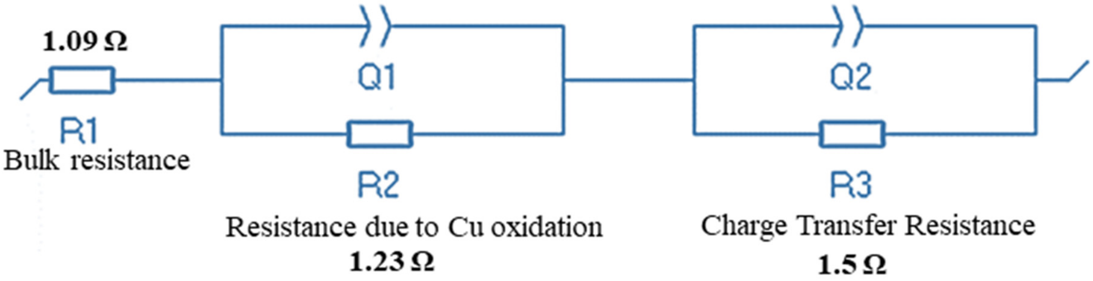

Figure S3. The equivalent circuit diagram fitted from the Nyquist data of Cu-O|Cu_3P.

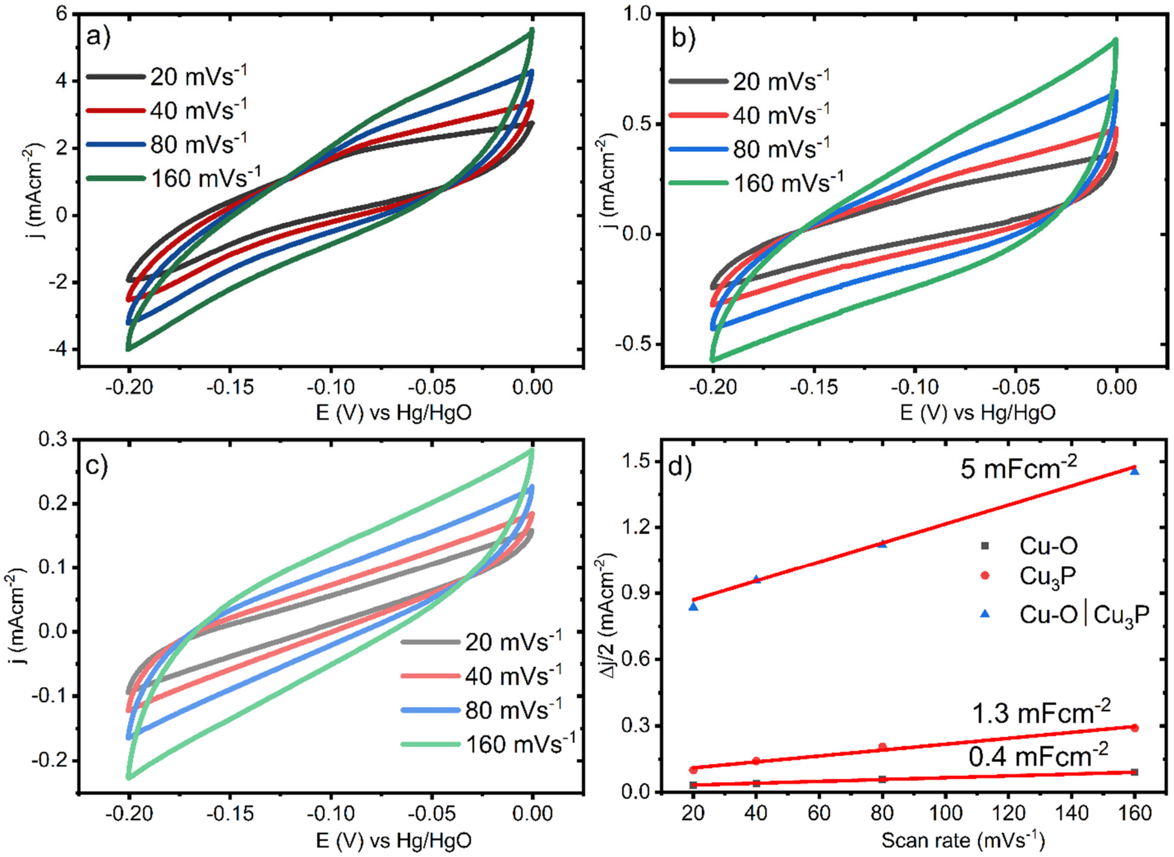

Figure S4. The cyclic voltammograms carried out at different sweep rates (**a**) Cu-O, (**b**) Cu_3P and (**c**) Cu-O|Cu_3P and (**d**) the double layer capacitance plots of three different catalysts.

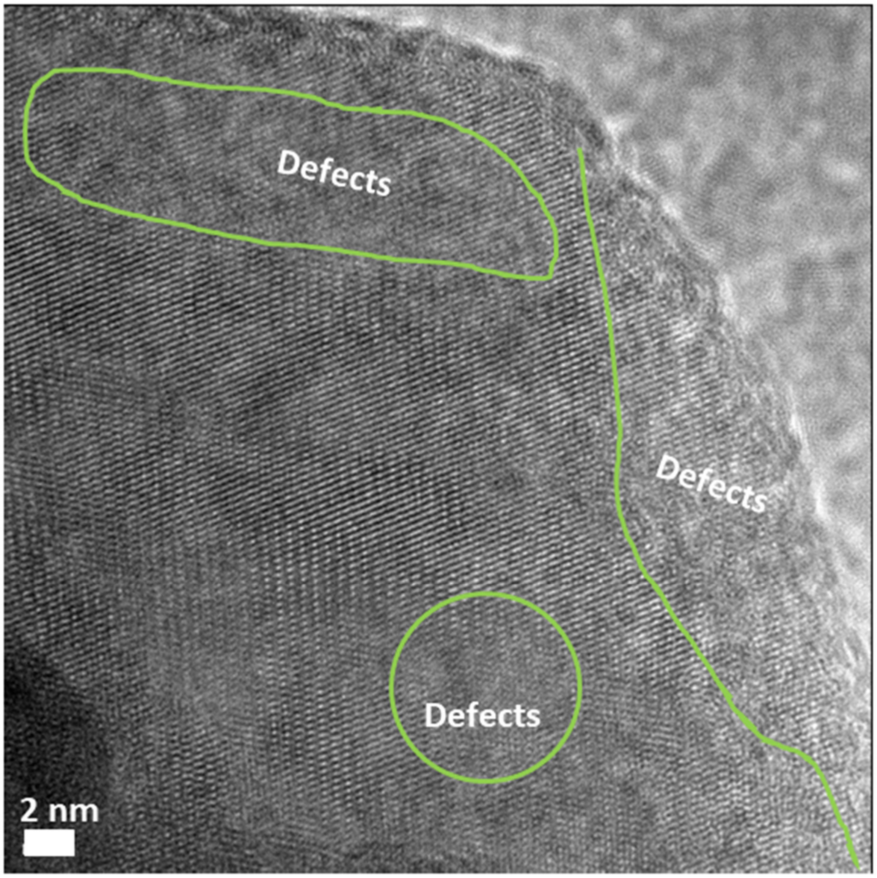

Figure S5. The HR-TEM of Cu-O|Cu_3P demonstrates the defects and randomness of the planes.

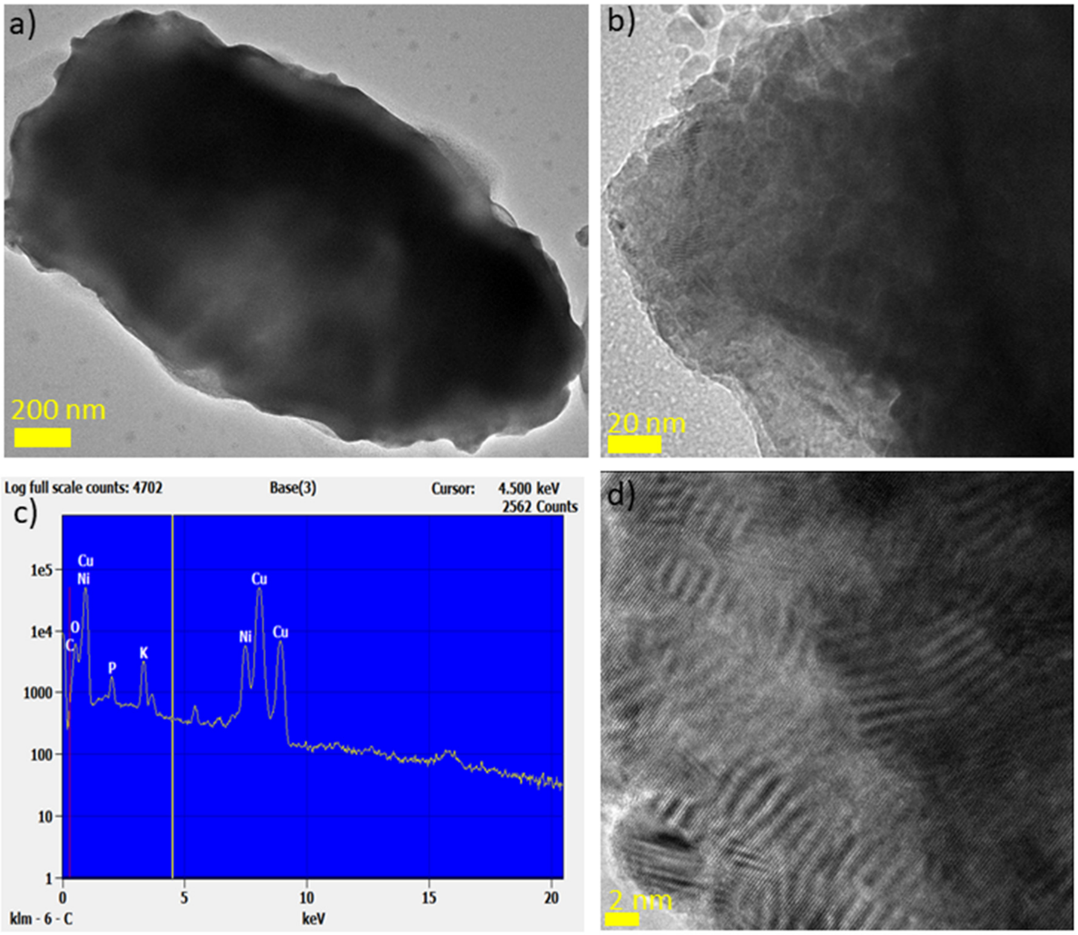

Figure S6. (**a**) the morphology of Cu-O|Cu_3P after MOR, (**b**) the high-resolution image after MOR, (**c**) demonstrates the disorderness of the planes after MOR and (**d**) EDS of the post MOR sample to notice the presence of various elements.
